# Predicting the risk of Chronic Kidney Disease in Men and Women in England and Wales: prospective derivation and external validation of the QKidney^® ^Scores

**DOI:** 10.1186/1471-2296-11-49

**Published:** 2010-06-21

**Authors:** Julia Hippisley-Cox, Carol Coupland

**Affiliations:** 1 Division of Primary Care, 13th floor, Tower Building, University Park, Nottingham, NG2 7RD, UK

## Abstract

**Background:**

Chronic Kidney Disease is a major cause of morbidity and interventions now exist which can reduce risk. We sought to develop and validate two new risk algorithms (the QKidney^® ^Scores) for estimating (a) the individual 5 year risk of moderate-severe CKD and (b) the individual 5 year risk of developing End Stage Kidney Failure in a primary care population.

**Methods:**

We conducted a prospective open cohort study using data from 368 QResearch^® ^general practices to develop the scores. We validated the scores using two separate sets of practices - 188 separate QResearch^® ^practices and 364 practices contributing to the THIN database.

We studied 775,091 women and 799,658 men aged 35-74 years in the QResearch^® ^derivation cohort, who contributed 4,068,643 and 4,121,926 person-years of observation respectively.

We had two main outcomes (a) moderate-severe CKD (defined as the first evidence of CKD based on the earliest of any of the following: kidney transplant; kidney dialysis; diagnosis of nephropathy; persistent proteinuria; or glomerular filtration rate of < 45 mL/min) and (b) End Stage Kidney Failure.

We derived separate risk equations for men and women. We calculated measures of calibration and discrimination using the two separate validation cohorts.

**Results:**

Our final model for moderate-severe CKD included: age, ethnicity, deprivation, smoking, BMI, systolic blood pressure, diabetes, rheumatoid arthritis, cardiovascular disease, treated hypertension, congestive cardiac failure; peripheral vascular disease, NSAID use and family history of kidney disease. In addition, it included SLE and kidney stones in women. The final model for End Stage Kidney Failure was similar except it did not include NSAID use.

Each risk prediction algorithms performed well across all measures in both validation cohorts. For the THIN cohort, the model to predict moderate-severe CKD explained 56.38% of the total variation in women and 57.49% for men. The D statistic values were high with values of 2.33 for women and 2.38 for men. The ROC statistic was 0.875 for women and 0.876 for men.

**Conclusions:**

These new algorithms have the potential to identify high risk patients who might benefit from more detailed assessment, closer monitoring or interventions to reduce their risk.

## Background

Chronic Kidney Disease (CKD) is a significant cause of morbidity and mortality across the developed world. It is associated with increased risk of death from cardiovascular disease[1-3], as well as from End Stage Kidney Failure[3]. The health burden due to CKD is likely to continue to rise with the ageing population and worldwide increase in Type 2 Diabetes[4]. CKD is an insidious disease characterised by a clear natural history with a detectable asymptomatic period during which interventions (such as blood pressure control) could help prevent or delay progression to End Stage Kidney Failure[5]. Despite this, patients are often identified and referred late when up to half already require Kidney dialysis or transplantation[6,7].

Whilst there are no randomised trials demonstrating that screening for CKD improves clinical outcomes, national programmes recommend that individuals who are at increased risk of CKD are tested for undetected kidney disease[8,9]. Reliable methods for identification of high risk patients are likely to be needed to identify and target early assessment and interventions to maximise health gain and improve outcomes[4].

High quality representative data within electronic patient records from primary care can be used to derive and validate robust risk prediction algorithms which can be then implemented and evaluated in clinical settings. This has already been achieved with the cardiovascular risk algorithm QRISK^®^2[10] which is integrated into the EMIS Clinic Computer System used by over half of general practices in the UK. The approach is being extended to other clinical conditions[11,12].

Although the case for developing a risk prediction model for CKD has been articulated[13], there are currently no widely accepted algorithms available to predict risk of CKD for an individual patient within primary care. In this paper, we describe the derivation and validation of two new risk predictions algorithms to predict 5 year risk of moderate-severe kidney disease. Designed to integrate with QRISK^®^2[10] and the QDScore^®^[12] for diabetes, the QKidney^® ^Scores complete the triad of prediction algorithms developed to identify patients at high risk of vascular disease for intervention.

## Methods

### Study design and sample

We did a prospective open cohort study in a large population of primary care patients using the QResearch^® ^database (version 22). We included all practices in England and Wales who had been using their EMIS computer system for at least a year. We used established methods for the study design and analysis which we summarise here and which are described and reviewed in detail elsewhere[10-12,14-16].

We randomly allocated two thirds of practices to the derivation cohort and the remaining third to a validation cohort. We identified an open cohort of patients aged 35-74 years without recorded evidence of CKD at study entry, registered with practices between the study start date of 01 Jan 2002 and the study end date of 31 Dec 2008. Patients entered the study on the latest of study start date, date of first registration with the practice or date they became 35 years old. Patients were censored at the earliest date of development of CKD, death, de-registration with the practice, last upload of computerised data or the study end date. Patients could therefore have up to 7 years of follow up data available.

We used the same inclusion and exclusion criteria to identify a separate sample of patients drawn from an independent set of practices contributing to the THIN primary care research database http://www.thin-uk.com except the study end date for this sample was 30 June 2008.

### Definition of Outcomes

We had two main outcomes. One outcome was recorded evidence of moderate-severe CKD defined as the first occurrence of any of the following during follow-up:

a) recorded kidney transplant;

b) recorded kidney dialysis;

c) recorded diagnosis of nephropathy;

d) glomerular filtration rate < 45 mL/min/1.73m^2 ^corresponding to stage 3B CKD[9].

e) recorded diagnosis of proteinuria;

Our second main outcome was End Stage Kidney Failure which was defined as the first occurrence of any of the following during follow-up:

a) recorded kidney transplant;

b) recorded kidney dialysis;

c) glomerular filtration rate < 15 mL/min/1.73m^2 ^corresponding to stage 5 CKD[9].

We calculated the glomerular filtration rates using the MDRD equation[17] using laboratory reported creatinine values

### Predictor variables

We developed models for men and women for each of our two main outcomes. Our initial list of predictor variables included those known to be associated with increased risk of CKD based on the literature[1,2,5,18,19] and from national guidance[9] which are also likely to be recorded in the patients electronic health record. We also sought the opinions of two senior nephrologists including the National Clinical Director for Kidney Services in England, Dr Donal O'Donoghue.

Variables examined for inclusion in both models were:

• Age at study entry (in single years)

• Body mass index

• Systolic blood pressure (mmHg)

• Smoking status (non-smoker, ex-smoker; light smoker: < 10 cigarettes/day, moderate smoker: 10-19 cigarettes per day, heavy smoker: 20 or more cigarettes per day)

• Ethnic group

• Townsend deprivation score (derived from the patient's postcode) [20]

• Diagnosis of type 1 diabetes

• Diagnosis of type 2 diabetes

• Diagnosis of cardiovascular disease

• Diagnosis of rheumatoid arthritis

• Treated hypertension

• Diagnosis of congestive cardiac failure

• Diagnosis of peripheral vascular disease

• Diagnosis of systemic lupus erythematosis

• Two or more prescriptions for NSAIDs drugs in the 6 months before study entry

• Evidence of kidney stones based on diagnosis or operative procedure at baseline

• Recorded family history of kidney disease including polycystic kidneys

• Prostatic hypertrophy at baseline (men)

In both models we only used diagnoses recorded prior to the baseline date as predictor variables, for body mass index, smoking status and systolic blood pressure we used the values recorded closest to the study entry date. Ethnic group was categorised as in previous publications[10].

### Statistical analysis

We developed and validated the risk prediction algorithms using established methods described in detail elsewhere [10-12,14-16]. In summary, we used Cox's proportional hazards models to estimate the coefficients for each risk factor for both outcomes for men and women separately, adjusting for other baseline risk factors. We excluded patients with the outcome at baseline. We used fractional polynomials to model non-linear risk relationships with continuous variables[21]. We compared models using the Akaike information criterion (AIC). We used multiple imputation to replace missing values for body mass index, systolic blood pressure and smoking status and used these values in our main analyses[22-25]. We carried out 5 imputations. We examined interactions between predictor variables and age, and we included significant variables and significant interaction terms in the final models. We took the regression coefficients for each variable from the final models and used these as weights which we combined with the baseline survivor function for moderate-severe CKD evaluated at 5 years to derive risk equations for (a) moderate-severe CKD and (b) End Stage Kidney Failure at 5 years' follow-up.

We applied the algorithms obtained from the derivation cohort to both validation cohorts and calculated measures of discrimination (D statistic[26] , R^2 ^statistic for survival data and area under the receiver operating characteristic curve (ROC statistic)) and calibration (comparing observed with predicted risks by tenth of predicted risk). We used the THIN validation sample for our main validation as this is from practices using a different clinical computer system from QResearch practices. We used all the available data on each database to maximise the power and also generalisability of the results. We used STATA (version 11) for all analyses.

The project has been independently reviewed in accordance with the QResearch^® ^agreement with Trent Multi-Centre Research Ethics Committee.

## Results

### Study population

Overall, 556 QResearch^® ^practices in England and Wales met our inclusion criteria, of which 368 were randomly assigned to the derivation dataset and 188 to the QResearch^® ^validation dataset.

In the QResearch^® ^derivation cohort there were 1,591,884 patients aged 35 to 74 at baseline of whom 17,135 had recorded evidence of pre-existing CKD and were therefore excluded leaving 1,574,749 patients for analysis. Of those with pre-existing CKD, 1,266 women and 1,524 men had End Stage Kidney Failure. In the QResearch^® ^validation cohort there were 796,598 patients aged 35 to 74 at baseline of whom 8,278 had recorded evidence of CKD at baseline leaving 788,320 for analysis.

There were 364 practices from the THIN database which met our inclusion criteria. The THIN cohort consisted of 1,595,141 patients aged 35 to 74 of whom 13,396 had recorded evidence of CKD at baseline leaving 1,581,745 for analysis.

Baseline characteristics for both the THIN and QResearch^® ^validation cohorts were very similar to the QResearch^® ^derivation cohort in both men and women as shown in Table 1.

**Table 1:** Characteristics of patients aged 35-74 years free of CKD at baseline in the QResearch derivation and the THIN validation cohort.

	*QResearch* *derivation cohort* *(n = 1,574,749)*	*THIN* *Validation cohort* *(n = 1,581,745)*
Women	775,091 (49.22)	784005 (49.57)
Mean age(SD)	47.3 (11.1)	49 (11.1)
Mean Townsend deprivation score (SD)	-0.5 (3.4)	-0.7 (3.1)
**Ethnic group**		
white/not recorded	1,511,028 (95.95)	1,548,749 (97.91)
Indian	12,360 (0.78)	8,313 (0.53)
Pakistani	6,582 (0.42)	2,370 (0.15)
Bangladeshi	2,671 (0.17)	797 (0.05)
Other Asian	5,239 (0.33)	3,652 (0.23)
Black Caribbean	9,596 (0.61)	4,131 (0.26)
Black African	10,535 (0.67)	5,224 (0.33)
Chinese	2,819 (0.18)	1,279 (0.08)
Other	13,919 (0.88)	7,230 (0.46)
**Smoking status**		
non smoker	804,831 (51.11)	641,929 (40.58)
ex smoker	286,731 (18.21)	236,820 (14.97)
light smoker	101,273 (6.43)	96,347 (6.09)
moderate smoker	124,246 (7.89)	153,262 (9.69)
heavy smoker	100,891 (6.41)	150,036 (9.49)
smoker amount not recorded	42,309 (2.69)	204,564 (12.93)
smoking not recorded	114,468 (7.27)	98,787 (6.25)
**Clinical conditions**		
Type 1 diabetes	4,441 (0.28)	4,940 (0.31)
Type 2 diabetes	49,179 (3.12)	49,877 (3.15)
Cardiovascular disease	70,865 (4.50)	80,977 (5.12)
Congestive cardiac failure	7,366 (0.47)	8,073 (0.51)
Peripheral vascular disease	16,015 (1.02)	20,066 (1.27)
Treated hypertension	156,506 (9.94)	142,192 (8.99)
Rheumatoid arthritis	11,933 (0.76)	14,137 (0.89)
Systemic lupus erythematosis	1,094 (0.07)	1,103 (0.07)
Kidney Stones	10,674 (0.68)	12,115 (0.77)
Current use of NSAIDS	425,775 (27.04)	387,087 (24.47)
Family history of kidney disease	719 (0.05)	1,097 (0.07)
**Clinical values**		
systolic blood pressure recorded	1,415,467 (89.89)	1,437,893 (90.91)
mean systolic blood pressure (SD)	133.1 (19.5)	133.6 (19.6)
body mass index recorded	1,241,158 (78.82)	1,253,686 (79.26)
mean body mass index (SD)	26.7 (4.7)	26.8 (4.7)
creatinine recorded ever	880,627 (55.92)	863,948 (54.62)
mean Creatinine (SD)	86.2 (15.9)	86.3 (16.3)
creatinine recorded prior to baseline	331,018 (21.02)	217,814 (13.77)
complete data (for SBP, BMI & smoking)	1,214,906 (77.15)	1,226,974 (77.57)

Values are numbers (%).

Data for the QResearch validation cohort is not shown but is available from the authors.

### Missing data

Table 1 also shows the proportions of patients with values recorded for smoking status, body mass index, systolic blood pressure and serum creatinine. Complete data for smoking status, body mass index and systolic blood pressure were available for 77.15% of patients in the QResearch derivation cohort and 77.57% of patients in the THIN validation cohort. There were differences in observed characteristics between those with and without missing data. As in previous studies[12,27], this pattern of missing data supports the use of multiple imputation (results available from the authors) under the assumption that data are missing at random.

### Incidence rates for both outcomes

Table 2 shows the age/sex incidence rates of moderate-severe CKD and Table 3 shows the incidence rates for End Stage Kidney Failure in both the QResearch^® ^derivation cohort and the THIN validation cohort.

**Table 2:** Incidence rates of moderate-severe CKD per 10,000 person years (95% CI) in the QResearch derivation cohort and THIN validation cohort

		*QResearch* *derivation cohort*	*THIN* *validation cohort*
**Women**	**Total**	**58.46 (57.72 to 59.21)**	**64.61 (63.81 to 65.43)**
	**35-39 yrs**	5.27 (4.76 to 5.83)	5.57 (5.02 to 6.17)
	**40-44 yrs**	7.11 (6.49 to 7.79)	9.34 (8.59 to 10.15)
	**45-49 yrs**	14.09 (13.13 to 15.11)	14.62 (13.62 to 15.70)
	**50-54 yrs**	24.35 (23.07 to 25.70)	26.61 (25.23 to 28.07)
	**55-59 yrs**	43.76 (42.02 to 45.56)	47.98 (46.11 to 49.93)
	**60-64 yr**s	89.55 (86.70 to 92.50)	99.94 (96.83 to 103.14)
	**65-69 yrs**	167.61 (163.41 to 171.92)	182.76 (178.22 to 187.43)
	**70-74 yrs**	291.44 (285.44 to 297.57)	315.58 (309.13 to 322.16)
			
**Men**	**Total**	**42.05 (41.43 to 42.68)**	**49.81 (49.10 to 50.52)**
	**35-39 yrs**	4.61 (4.15 to 5.12)	6.26 (5.69 to 6.89)
	**40-44 yrs**	6.82 (6.23 to 7.47)	9.92 (9.17 to 10.74)
	**45-49 yrs**	11.7 (10.85 to 12.61)	15.72 (14.70 to 16.82)
	**50-54 yrs**	18.82 (17.71 to 19.99)	24.06 (22.76 to 25.44)
	**55-59 yrs**	33.47 (31.96 to 35.04)	40.93 (39.21 to 42.73)
	**60-64 yrs**	65.64 (63.19 to 68.19)	75.49 (72.77 to 78.31)
	**65-69 yrs**	129.01 (125.23 to 132.90)	150.21 (145.98 to 154.56)
	**70-74 yrs**	224.47 (218.85 to 230.24)	248.59 (242.41 to 254.93)

**Table 3:** Incidence rates of End Stage Kidney Failure per 10,000 person years (95% CI) in the QResearch derivation cohort and THIN validation cohort

		*QResearch* *derivation cohort*	*THIN* *validation cohort*
**Women**	**total**	**3.03 (2.87 to 3.20)**	**3.03 (2.86 to 3.21)**
	**35-39 yrs**	0.83 (0.65 to 1.07)	0.70 (0.52 to 0.93)
	**40-44 yrs**	0.95 (0.74 to 1.22)	1.18 (0.93 to 1.49)
	**45-49 yrs**	1.40 (1.12 to 1.74)	1.41 (1.13 to 1.78)
	**50-54 yrs**	1.64 (1.33 to 2.02)	1.95 (1.61 to 2.38)
	**55-59 yrs**	2.56 (2.17 to 3.02)	2.99 (2.55 to 3.50)
	**60-64 yrs**	4.45 (3.86 to 5.13)	4.29 (3.69 to 4.98)
	**65-69 yrs**	6.44 (5.68 to 7.30)	7.00 (6.18 to 7.93)
	**70-74 yrs**	11.6 (10.52 to 12.79)	9.59 (8.58 to 10.72)
			
**Men**	**Total**	**3.66 (3.48 to 3.85)**	**3.88 (3.69 to 4.08)**
	**35-39 yrs**	1.01 (0.80 to 1.26)	1.19 (0.95 to 1.48)
	**40-44 yrs**	1.18 (0.95 to 1.47)	1.55 (1.27 to 1.89)
	**45-49 yrs**	1.51 (1.22 to 1.86)	1.98 (1.64 to 2.39)
	**50-54 yrs**	2.30 (1.94 to 2.73)	2.38 (2.00 to 2.84)
	**55-59 yrs**	3.64 (3.17 to 4.18)	3.58 (3.10 to 4.14)
	**60-64 yrs**	5.09 (4.44 to 5.82)	5.22 (4.55 to 5.99)
	**65-69 yrs**	9.48 (8.52 to 10.55)	9.71 (8.70 to 10.83)
	**70-74 yrs**	14.24 (12.93 to 15.68)	14.36 (12.98 to 15.88)

During the 4,068,643 person years of follow up for women in the derivation cohort without moderate-severe CKD at baseline, there were 23,786 incident cases of moderate-severe CKD giving an overall incidence rate of 58.46 per 10,000 person years. For men, there were 17,333 moderate-severe CKD cases arising from 4,121,926 person years giving a crude incidence rate of 42.05 per 10,000 person years. During the 4,177,287 person years of follow up for women in the derivation cohort without End Stage Kidney Failure at baseline, there were 1,266 incident cases of End Stage Kidney Failure giving an overall incidence rate of 3.03 per 10,000 person years. For men, there were 1,534 cases of End Stage Kidney Failure arising from 4,193,578 person years giving a crude incidence rate of 3.66 per 10,000 person years.

The incidence rates for both outcomes in the THIN validation cohort were very similar to that for both QResearch^® ^cohorts.

### Model Development

#### Moderate-severe CKD

Table 4 shows the variables included in the final algorithm for moderate-severe CKD with the hazard ratios, fractional polynomial terms for the continuous variables and the associated interaction terms. The highest risks of moderate-severe CKD occurred with Type 1 diabetes (adjusted hazard ratio 12.30, 95% CI 10.3 to 14.6 for men and 8.21, 95% CI 6.74 to 9.99 for women). The adjusted hazard ratios were lower for Type 2 diabetes (6.07, 95% CI 5.61 to 6.57 for men and 4.50, 95% CI 4.14 to 4.89 for women).

**Table 4:** Adjusted hazard ratios for risk of moderate-severe CKD using the QResearch derivation cohort

	***Adjusted hazard ratio*** ***(95%CI)*** ***women***	***Adjusted hazard ratio*** ***(95%CI)*** ***men***
**Ethnic group**		
White or ethnicity not recorded		
Indian^§^	1.03 (.896 to 1.18)	1.16 (1.01 to 1.34)
Pakistani^§^	1.55 (1.32 to 1.81)	2.00 (1.70 to 2.35)
Bangladeshi^§^	1.50 (1.14 to 1.95)	1.35 (1.03 to 1.78)
Other Asian^§^	1.16 (0.89 to 1.52)	1.44 (1.09 to 1.90)
Black Caribbean^§^	0.48 (0.41 to 0.57)	0.83 (0.69 to 0.99)
Black African^§^	0.56 (0.43 to 0.74)	1.17 (0.90 to 1.54)
Chinese^§^	1.13 (0.77 to 1.65)	1.36 (0.89 to 2.07)
Other ethnic group^§^	1.23 (1.07 to 1.40)	1.33 (1.13 to 1.57)
		
**Co-morbidity**		
Type 1 diabetes ^±^	8.21 (6.74 to 9.99)	12.3 (10.3 to 14.6)
Type 2 diabetes ^±^	4.50 (4.14 to 4.89)	6.07 (5.61 to 6.57)
Cardiovascular disease ^±^	1.37 (1.32 to 1.42)	1.40 (1.34 to 1.45)
Congestive cardiac failure ^±^	2.27 (2.11 to 2.45)	2.84 (2.66 to 3.03)
Peripheral vascular disease	1.35 (1.25 to 1.46)	1.47 (1.37 to 1.57)
Treated hypertension ^±^	2.49 (2.33 to 2.66)	2.78 (2.59 to 2.99)
NSAID use ^±^	1.30 (1.27 to 1.34)	1.29 (1.25 to 1.33)
Family history of kidney disease ^±^	2.13 (1.39 to 3.27)	3.58 (2.19 to 5.84)
Rheumatoid arthritis ^±^	1.62 (1.51 to 1.75)	1.48 (1.30 to 1.67)
Systemic lupus erythematosis ^±^	2.40 (1.92 to 3.00)	n/a*
History of kidney stones ^±^	1.27 (1.11 to 1.46)	n/a*
**Smoking status**		
Non smoker	1.00	1.00
Ex smoker ^±^	1.19 (1.15 to 1.23)	1.13 (1.09 to 1.17)
Light smoker ^±^	1.30 (1.23 to 1.38)	1.15 (1.08 to 1.22)
Moderate smoker ^±^	1.27 (1.21 to 1.34)	1.24 (1.16 to 1.32)
Heavy smoker ^±^	1.43 (1.34 to 1.52)	1.25 (1.16 to 1.34)
		
Townsend score (per 5 unit increase)	1.16 (1.14 to 1.18)	1.10 (1.08 to 1.19)

^± ^compared with patients without the condition/medication at baseline.

^§ ^Compared with patients in the white/not recorded group

*n/a as not included in the final model

Models also included fractional polynomial terms for age, body mass index and systolic blood pressure. The fractional polynomial terms were as follows:

Both sexes: age terms were (age/10)^3 ^and (age/10)^3^*ln(age/10)

Both sexes: bmi terms were (bmi/10)^-2 ^and (bmi/10)^-2^*ln(bmi/10)

Women: sbp terms were (sbp/100)^-2 ^and (sbp/100)^-2^*ln(sbp/100)

Men: sbp terms were (sbp/100)^-2 ^and (sbp/100)^-0.5^

The model also included interactions between the age terms and type 1 diabetes, type 2 diabetes and treated hypertension

Pakistani patients had the highest risks which were almost twice those in the "White or ethnicity not recorded" group. The adjusted hazard ratios were 2.00 (95% CI 1.70 to 2.35) for Pakistani men and 1.55 (95% CI 1.32 to 1.81) for Pakistani women. Lowest risks were observed among black Caribbean women (adjusted hazard ratio 0.48, 95% CI 0.41 to 0.57) and Black African women (adjusted hazard ratio 0.56, 95% CI 0.43 to 0.74). Smokers, men and women from deprived areas, those with cardiovascular disease, congestive cardiac failure, peripheral vascular disease, NSAID use, family history of kidney disease, treated hypertension and rheumatoid arthritis also had increased risks compared with patients without those factors. Kidney stones and systemic lupus erythematosis were significant predictors in women but not in men. Prostatic hypertrophy was not an independent risk factor for men so was not included in the final model.

#### End Stage Kidney Failure

Table 5 shows the hazard ratios for End Stage Kidney Failure. Patients with type 1 and type 2 diabetes, congestive cardiac failure, treated hypertension and those with a family history of kidney disease all had more than twice the risk of End Stage Kidney Failure compared with patients without these factors. Kidney stones and systemic lupus erythematosis were significant predictors in women but not in men.

**Table 5:** Adjusted hazard ratios for risk of End Stage Kidney Failure using the QResearch derivation cohort

	*Adjusted hazard ratio* *(95%CI)* *women*	*Adjusted hazard ratio* *(95%CI)* *men*
**Ethnic group**		
White or ethnicity not recorded		
Indian^§^	1.16 (.691 to 1.94)	1.37 (0.90 to 2.07)
Pakistani^§^	2.48 (1.54 to 3.98)	1.83 (1.05 to 3.18)
Bangladeshi^§^	0.93 (0.30 to 2.90)	1.22 (0.54 to 2.75)
		
Other Asian^§^	3.07 (1.64 to 5.76)	2.39 (1.19 to 4.81)
Black Caribbean^§^	1.01 (0.64 to 1.61)	1.56 (1.01 to 2.43)
Black African^§^	1.63 (0.89 to 2.99)	1.87 (0.99 to 3.51)
Chinese^§^	3.50 (1.56 to 7.84)	insufficient numbers
Other ethnic group^§^	1.36 (0.82 to 2.23)	0.96 (0.52 to 1.79)
		
**Co-morbidity**		
Type 1 diabetes ^±^	22.3 (14.7 to 33.8)	11.3 (7.59 to 16.9)
Type 2 diabetes ^±^	4.68 (3.58 to 6.11)	2.79 (2.17 to 3.58)
Cardiovascular disease ^±^	1.35 (1.14 to 1.58)	1.34 (1.18 to 1.53)
Congestive cardiac failure ^±^	4.45 (3.55 to 5.57)	4.02 (3.33 to 4.86)
Peripheral vascular disease	1.7 (1.3 to 2.23)	1.98 (1.62 to 2.42)
Treated hypertension ^±^	4.8 (3.96 to 5.82)	6.77 (5.71 to 8.02)
Family history of kidney disease ^±^	6.41 (2.4 to 17.1)	9.68 (4.01 to 23.4)
Rheumatoid arthritis ^±^	1.52 (1.1 to 2.1)	1.53 (1.01 to 2.34)
Systemic lupus erythematosis ^±^	4.69 (2.63 to 8.35)	n/a
History of kidney stones ^±^	2.07 (1.34 to 3.19)	n/a
**Smoking status**		
Non smoker		
Ex smoker ^±^	1.22 (1.06 to 1.40)	1.16 (1.03 to 1.3)
Light smoker ^±^	1.45 (1.13 to 1.85)	1.17 (0.98 to 1.41)
Moderate smoker ^±^	1.08 (0.85 to 1.38)	1.33 (1.09 to 1.63)
Heavy smoker ^±^	1.43 (1.11 to 1.83)	1.09 (.858 to 1.38)
		
Townsend score (per 5 unit increase)	1.23 (1.13 to 1.34)	1.10 (1.02 to 1.19)

^± ^compared with patients without the condition/medication at baseline.

^§ ^Compared with patients in the white/not recorded group

Models also included fractional polynomial terms for age, body mass index and systolic blood pressure. The fractional polynomial terms were as follows:

Both sexes: age terms were (age/10)^3 ^and (age/10)^3^*ln(age/10)

Both sexes: bmi terms were (bmi/10)^-2 ^and (bmi/10)^-2^*ln(bmi/10)

Women: sbp terms were (sbp/100)^-2 ^and (sbp/100)^-2^*ln(sbp/100)

Men: sbp terms were (sbp/100)^-2 ^and (sbp/100)^-0.5^

The model also included interactions between the age terms and type 1 diabetes, type 2 diabetes and treated hypertension

Other predictors in both men and women included deprivation, smoking, cardiovascular disease, rheumatoid arthritis and peripheral vascular disease. The increased risks associated with these factors were less marked than the factors above. NSAID use was not significant in men or women. Prostatic hypertrophy was not an independent risk factor for men so was not included in the final model.

### Validation of the risk prediction algorithms

For the THIN cohort, the model to predict moderate-severe CKD explained 56.38% of the total variation in women and 57.49% for men (Table 6). The D statistic values were high indicating good discrimination with values of 2.33 for women and 2.38 for men. The ROC statistic was also high with values of 0.875 for women and 0.876 for men.

**Table 6:** Validation statistics for each models in the THIN and QResearch^® ^validation cohorts

		***moderate-severe CKD***	***End Stage Kidney Failure***
**Women**	**THIN cohort**		
	R^2 ^statistic (%)	56.19 (55.67 to 56.70)	54.02 (51.67 to 56.37)
	D statistic	2.32 (2.30 to 2.34)	2.22 (2.11 to 2.32)
	ROC statistic	0.875 (0.872 to 0.877)	0.818 (0.803 to 0.833)
			
	**QResearch cohort**		
	R^2 ^statistic (%)	56.45 (55.40 to 57.50)	55.39 (0.52.59 to 58.18)
	D statistic	2.33 (2.28 to 2.40)	2.28 (2.15 to 2.41)
	ROC statistic	0.877 (0.873 to 0.880)	0.843 (0.825 to 0.860)
			
**Men**	**THIN cohort**		
	R^2 ^statistic (%)	57.41 (54.56 to 60.27)	52.86 (50.55 to 55.17)
	D statistic	2.38 (2.24 to 2.51)	2.17 (2.07 to 2.27)
	ROC statistic	0.875 (0.873 to 0.878)	0.839 (0.827 to 0.850)
			
	**QResearch cohort**		
	R^2 ^statistic (%)	58.29 (55.31 to 61.26)	56.65 (53.94 to 59.35)
	D statistic	2.42 (2.28 to 2.56)	2.34 (2.21 to 2.47)
	ROC statistic	0.878 (0.874 to 0.882)	0.846 (0.829 to 0.862)

Notes on understanding validation statistics:

R^2 ^statistic shows explained variation - higher values indicate more variation is explained

ROC statistic is a measure of discrimination - higher values indicate better discrimination

D statistic is a measure of discrimination - higher values indicate better discrimination and an increase of 0.1 or more over other risk prediction models is a good marker of improved prognostic separation

The D statistic, R^2 ^statistic and ROC statistics had marginally higher values for the moderate-severe CKD model compared with the End Stage Kidney Failure model suggesting marginally better performance for the moderate-severe CKD model in both men and women (Table 6).

**Figure 1:**
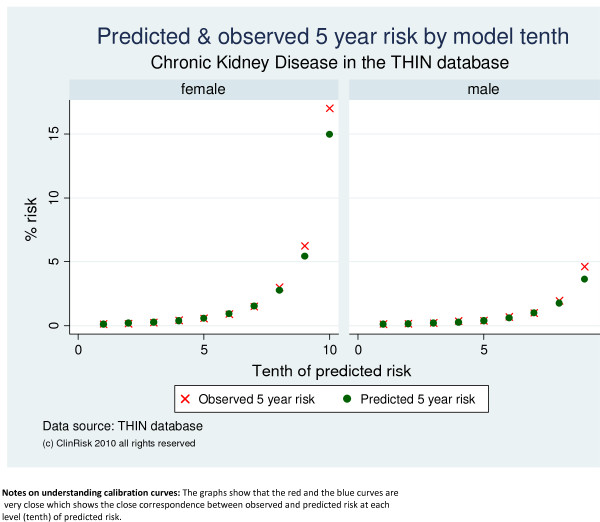
**Predicted and observed risks of moderate-severe CKD by model tenth in the THIN validation cohort**.

**Figure 2:**
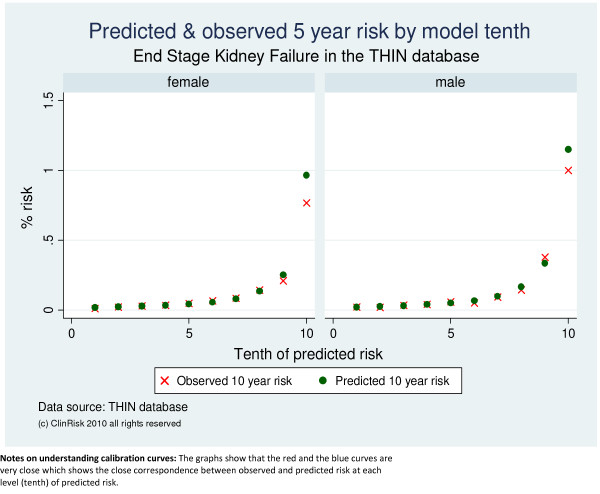
**Predicted and observed risks of End Stage Kidney Failure by model tenth in the THIN validation cohort**.

Both algorithms were well calibrated in the THIN cohort as shown by the close correspondence between observed and predicted values in the calibration graphs across tenths of predicted risk both for moderate-severe CKD (Figure 1) and End Stage Kidney Failure (Figure 2).

The corresponding results for all validation statistics for both models in men and women in the QResearch validation cohort were very similar as can be seen in Table 6.

### Clinical assessment tools

#### Individual clinical assessment

**Figure 3:**
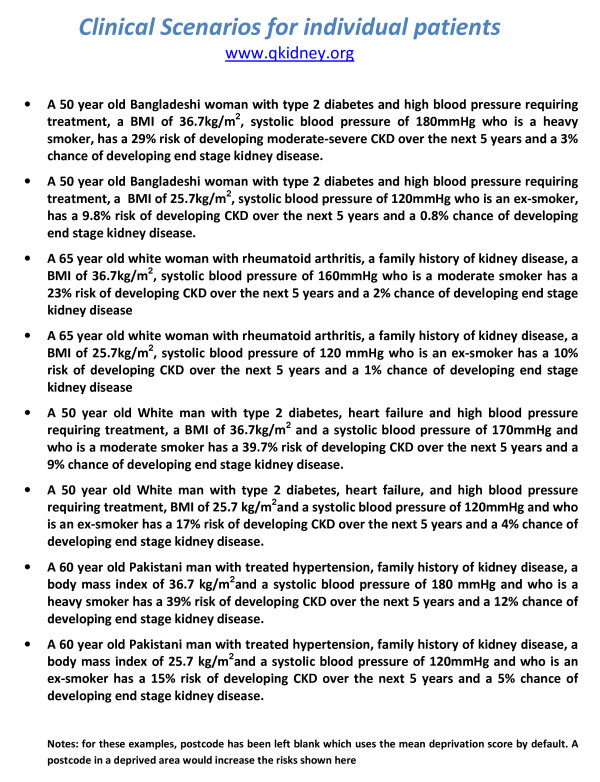
**Clinical case histories for individual patients using the Web Calculator **. http://www.qkidney.org.

Figure 3 shows 8 clinical case histories using the model to predict moderate-severe CKD and end stage kidney failure using the web calculator http://www.qkidney.org

#### Population assessment using the risk stratification tool

The QKidney^® ^Scores can also be used to risk stratify an entire population aged 35-74 years to identify patients at highest risk for more proactive intervention as part of the systematic Vascular Risk Assessment Programme currently underway in the UK[8]. Since there are no established thresholds for risk of CKD comparable with the 20% threshold for cardiovascular disease[28-30], we defined these based on the distribution of the models within the QResearch validation cohort (which were extremely similar to those also found in the THIN analysis)

For example, the cut off for the top tenth for risk of moderate-severe CKD gives a 5 year risk threshold of 5.46% in men and 8.01% in women. This top tenth contained 58.01% of all men in the QResearch validation cohort who developed moderate-severe CKD over the 5 year period from baseline and 55.68% of women.

For End Stage Kidney Failure, the cut off for the top tenth gives a 5 year risk threshold of 0.49% in men and 0.36% in women. This top tenth contained 55.93% of all men in the QResearch validation cohort who developed End Stage Kidney Failure over the 5 year period from baseline and 55.44% of women.

Applying the QResearch age/sex incidence rates of moderate-severe CKD to the estimated population of England and Wales aged 35-74 for 2007, we estimate there will be about 807,400 new cases of moderate-severe CKD in the next 5 years. This is a conservative estimate since the population is likely to age over the next 5 years. Using the moderate-severe CKD algorithm to identify the 10% of patients with the highest risk would be expected to identify approximately 457,700 of the patients who develop moderate or severe CKD over the next 5 years. Assuming an intervention with 10% effectiveness at reducing risk, then approximately 45,770 cases of CKD could be prevented in England and Wales over the next 5 years by targeting the intervention at those at greatest risk.

Applying the QResearch age/sex incidence rates of End Stage Kidney Failure to the estimated population of England and Wales aged 35-74 for 2007, we estimate there will be about 46,500 new cases of End Stage Kidney Failure in the next 5 years. Using the End Stage Kidney Failure algorithm to identify the 10% of patients with the highest risk, would identify approximately 25,900 of these patients. Assuming an intervention with 10% effectiveness at reducing risk, then approximately 2,590 cases of End Stage Kidney Failure could be prevented in England and Wales over the next 5 years by targeting the intervention at those at greatest risk.

## Discussion

### Summary of Main Findings

We have derived and validated two new algorithms designed to predict an individual's 5 year risk of being diagnosed with (a) moderate-severe CKD or (b) End Stage Kidney Failure. Both algorithms are based on factors which the user is likely to know and which are likely to be recorded within the patient's electronic health record. The algorithms do not require a laboratory measurement. They are therefore suitable for situations where this information is not readily available and can be used as part of a Vascular Risk Assessment to flag up those patients who need referral to the GP for a more detailed assessment. They can also be used to inform patients about their level of absolute risk to help them make an informed choice regarding the need for further assessment or intervention.

At population level, these algorithms can be used to "risk stratify" the entire population to systematically identify those patients who need investigation (e.g. creatinine test) or further assessment or regular monitoring. This could be achieved by automatically applying these algorithms to the computerised medical records of all patients aged 35-74 registered with a practice. This meets a core requirement of the NHS Programme for IT, namely to "calculate the risk of the renal function deteriorating, taking all recorded risk factors into account, and the recalculation of risk on a regular basis to take account of changes as a result of ageing or whenever more patient information becomes available (e.g. test results)" (personal communication).

Once identified, high risk patients can then avoid nephrotoxic drugs (such as NSAIDs), have more energetic treatment to lower blood pressure, reduce blood pressure targets or have more frequent follow up of kidney function to allow earlier referral to secondary care services. Further research is required to identify the effectiveness of these interventions in a high risk population.

### Comparison with other studies

To our knowledge, these are the first algorithms to predict both the risk of moderate-severe CKD and the risk of End Stage Kidney Failure in UK primary care. They improve on a recently described algorithm to predict CKD derived using 9,470 participants from the American ARIC/CHS cohorts [31]. Both studies used similar statistical methods for the derivation and validation of the algorithms. In the ARIC/CHS study, the outcome included patients with the more mild Stage 3a disease which has less certain prognostic significance. Our algorithms include additional known risk factors such as family history of kidney disease, use of NSAIDS, kidney stones, rheumatoid arthritis, systemic lupus erythematosis, body mass index and smoking status. They also include more detailed variables for ethnic group, interactions with age and distinguish between type 1 and type 2 diabetes, which have markedly different risks. Our ROC values were all in excess of 0.82 which is substantially better than the ROC statistic of 0.70 reported in the ARIC/CHS study[31].

### Strengths and weaknesses

Strengths and weaknesses of our study are likely to be similar to those discussed in detail elsewhere[10-12,14-16]. Weaknesses, as with all observational studies, include the potential for bias. Misclassification bias of outcome or predictor variables could have occurred, which, if non-differential would tend to bias the hazard ratios towards one and reduce discrimination. However, it is probable that patients with established risk factors such as diabetes would be more likely to have blood or urine tests and this could have the effect of inflating hazard ratios associated with these risk factors. Nonetheless, our hazard ratios for the risk factors in the model apart from diabetes, are generally of a similar magnitude to those found in other similar studies which tested for chronic kidney disease in the entire study cohort[31]. In addition, the assessment and recording of these factors in clinical practice is becoming increasingly routine and complete, so limiting the effect of this potential bias.

Whilst the outcomes were not adjudicated by a panel of clinicians, we think it unlikely that more than a small number of patients will have been misclassified as having the outcomes since the definitions are based on objective measurements or major operations or procedures. It is possible, indeed likely, that some patients had undetected or un-recorded kidney disease at baseline or follow-up since there is no systematic widespread testing of blood or urinalysis. This is in fact part of the justification for a systematic population based approach.

We have based the date of our outcome on the date of first recorded evidence of moderate-severe kidney disease and of end stage kidney failure. Given the insidious and gradual nature of decline in kidney function, it is likely that the real onset occurred before the date of the recorded onset. This will have tended to result in a general under-estimation of incidence rates which in turn would lead to an under-estimation in individualised risk estimates. However, we think in some cases the date of first recorded evidence may relate closely to the onset of symptoms leading to a consultation and blood or urine tests. Although some alternative analytical methods are available to allow for interval censored data these tend to make stronger assumptions than Cox regression about the distribution of the hazard rate, and often group all outcome dates into fixed intervals, hence potentially losing precision.

Key strengths include size, representativeness due to inclusion of entire practice populations and quality of the database used to derive the algorithm and its ability to generalise back into the setting where it can be applied. The algorithms are well calibrated to the setting in which they can be used and have good levels of discrimination. Our study has good face validity as the vast majority of risk factors identified in the literature or by consensus were found to be independent predictors and hence included in the QKidney^® ^Scores[1,2,18]. As in other studies, we found an association between increasing levels of deprivation and risk of CKD [3] as well as confirming ethnic differences [19]. The inclusion of ethnic group and deprivation within the risk prediction scores should help avoid widening inequalities which can occur at the start of major new public health initiatives[32]. Lastly, we have validated each algorithm in an external set of practices contributing to the THIN database and demonstrated comparable performance with the validation cohort from the QResearch^® ^database. We found very close similarities for a wide range of population characteristics between the THIN and QResearch^® ^cohorts. This helps validate both databases and is reassuring regarding the likely generalisability of results from research using either database to the rest of the UK.

## Conclusions

We have developed and validated two new risk prediction algorithms designed to predict risk of moderate-severe CKD and End Stage Kidney Failure in primary care which have the potential to identify high risk patients who might benefit from more detailed assessment, closer monitoring or interventions to reduce their risk. Further research is required to verify these findings as well as to identify the effectiveness of using this approach with appropriate interventions in a high risk population.

## Competing interests

JHC is co-director of QResearch^® ^- a not-for-profit organisation which is a joint partnership between the University of Nottingham and EMIS (leading commercial supplier of IT for 60% of general practices in the UK). EMIS may implement the QKidney^® ^Scores within its clinical system. JHC is also director and co-founder of ClinRisk Ltd which produces open and closed software to ensure the reliable and updatable implementation of clinical risk algorithms within clinical computer systems. JHC is also a general practitioner and professor of clinical epidemiology at the University of Nottingham. CC is associate professor of Medical Statistics at the University of Nottingham and a consultant statistician for ClinRisk Ltd. This work and any views expressed within it are solely those of the co-authors and not of any affiliated bodies or organisations.

## Authors' contributions

JHC initiated the study, undertook the literature review, data extraction, data manipulation and primary data analysis and wrote the first draft of the paper. CC contributed to the design, analysis, interpretation and drafting of the paper. Both authors read and approved the final version of the manuscript.

## Authors information

JHC is professor of Clinical Epidemiology & General Practice at the University of Nottingham (UK). CC is associate professor of Medical Statistics at the University of Nottingham (UK).

## Funding

The work was undertaken by ClinRisk. There was no external funding.

## Pre-publication history

The pre-publication history for this paper can be accessed here:

http://www.biomedcentral.com/1471-2296/11/49/prepub
